# Safety and efficacy concerns in Indian probiotics: Insights from whole-genome sequencing and *in vitro* assessment

**DOI:** 10.1080/29933935.2026.2668863

**Published:** 2026-05-14

**Authors:** Irulappan Madhumathi, Vishnukumar Ramesh, Boomiga Rathakrishnan, Baby Abirami, Karthik Gunasekaran, T. Sathish Kumar, Priyadharshini Jeyasankar, Gunachandru Raja, Karnan Selvam, Subbulakshmi Rajendran, Ayyanraj Neeravi, Kamini Walia, Balaji Veeraraghavan

**Affiliations:** a Department of Clinical Microbiology, Christian Medical College, Vellore, India; b Department of General Medicine, Christian Medical College, Vellore, India; c Department of Paediatrics, Christian Medical College, Vellore, India; d Division of Descriptive Research, Indian Council of Medical Research, New Delhi, India

**Keywords:** Probiotics, acute diarrhea, *Saccharomyces boulardii*, antimicrobial resistance, quality control, India, regulatory oversight

## Abstract

Acute diarrhea remains a major public health challenge in India, particularly among children, where probiotics are commonly used as adjunctive therapy. However, concerns regarding the quality, safety, and regulatory oversight of commercial probiotic formulations persist. This study evaluated 13 probiotic products marketed in India for antibiotic susceptibility, gastrointestinal survivability, and safety. A combined *in vitro* and genomic approach was employed, including microbial enumeration, MALDI-TOF identification, MIC testing, time-kill assays, simulated gastrointestinal survival, and whole-genome sequencing to assess strain identity, antimicrobial susceptibility, antimicrobial resistance genes (ARGs), and virulence-associated determinants. Five of the 13 products showed discrepancies between labeled and laboratory-enumerated CFU counts, and some contained contaminants such as *Enterococcus faecium*. Several bacterial probiotics used outdated nomenclature (e.g., “LAB” or *Lactobacillus sporogenes*), inconsistent with current taxonomy. Bacterial probiotics were found to be susceptible to co-prescribed antibiotics, which may compromise their therapeutic persistence. Whole-genome sequencing identified multiple antimicrobial resistance determinants, including *tet(L), erm(34), blaBCL-1*, *catA10*, and *ant(4′)-Ib*. Among these, *tet(L), catA10,* and *ant(4′)-Ib* showed higher predicted potential for mobilization, supported by literature and the presence of insertion sequence elements in this study. In contrast, *erm(34)* and *blaBCL-1* are generally considered chromosomally encoded intrinsic resistance determinants. In contrast, the yeast probiotic *Saccharomyces boulardii* is intrinsically resistant to antibacterial agents and shows superior gastrointestinal survivability. Overall, inconsistencies in labeling, composition, and genomic safety underscore the need for strain-level labeling, genomic validation, and stronger regulatory oversight to ensure probiotic quality and safety.

## Summary

- **What is already known on this topic?**
  Probiotics are commonly used to manage acute diarrhea, especially in children. Specific strains, such as *Saccharomyces boulardii*, *Lactobacillus rhamnosus* GG, and *Bacillus clausii,* have shown clinical benefits. However, the quality, strain identity, and antibiotic susceptibility of commercial probiotics remain questionable. In India, weak regulatory oversight permits vague labeling and limited quality control, despite the existence of non-mandatory ICMR-DBT guidelines.
- **What this study adds?**
  This is the first comprehensive *in vitro* and genomic assessment of 13 probiotic products sold in India. The study reveals widespread issues with strain mislabeling, contamination, antibiotic resistance genes (ARGs), and poor gastrointestinal survivability. Only *S. boulardii* 745 (Econorm) showed consistent viability, genomic safety, and antibiotic resilience, making it the most reliable option for diarrhea treatment.
- **How this study might affect research, practice, or policy?**
  The findings urge caution in co-prescribing bacterial probiotics with antibiotics due to variable resistance and safety concerns. *S. boulardii* is reinforced as the preferred probiotic for diarrhea, particularly in high-risk patients. These results support the routine use of genomic tools in product evaluation. To ensure safety and efficacy, policy reforms must prioritize strain-specific labeling, genomic validation, and mandatory premarket testing.

## Graphical abstract

**Figure:**
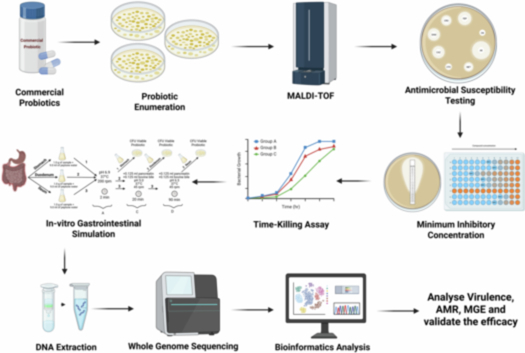

## Introduction

Acute diarrhea remains a significant global health concern, particularly among children under 2 y of age. It is caused by pathogens like rotavirus, *Salmonella*, *Shigella*, *Escherichia coli*, and *Vibrio cholerae*, antibiotics-associated *Clostridioides difficle* infections. In addition, non-infectious factors like antibiotics, malnutrition, and food hypersensitivity also lead to diarrhea^1^^,^^2^ that can lead to serious dehydration and malnutrition if not treated.

Probiotics, as defined by the Food and Agriculture Organization of the United Nations (FAO) and the World Health Organization (WHO), are live microorganisms that confer health benefits in adequate doses.^3^ They help treat acute diarrhea by restoring gut flora, strengthening mucosal barriers, and modulating immunity.^1^^,^^4^ Key strains like *Lactobacillus rhamnosus* GG, *Bifidobacterium*, *Bacillus* spp., and *Saccharomyces cerevisiae* boulardii are given at 5–20 billion CFUs/d based on age.^4^^,^^5^ Strain-specific formulations (e.g., capsules, sachets) are standardized for mass production, though patenting remains challenging.^3^

However, bacterial probiotics are often sensitive to commonly used antibiotics (e.g., penicillin, tetracycline, and ofloxacin), limiting their viability during therapy.^6^^,^^7^ Though *Bacillus* species has been shown greater resilience due to spore formation, strain-dependent susceptibility can undermine their efficacy.^8^ Strategies such as strain-specific selection and timing of administration (2–4 hours apart) help mitigate these effects.

In contrast, yeast probiotics like *Saccharomyces boulardii* are inherently antibiotic-resistant and effective against *C. difficile* and *Helicobacter pylori*. Their robust survival in gastric conditions supports their clinical use, especially in antibiotic-associated diarrhea.^4^ Clinical trials supports its efficacy in reducing the duration and severity of acute diarrhea (e.g., NCT03539913), especially in pediatric populations.^9^

Despite rising probiotic use, regulatory inconsistencies; including vague classification, lack of standardized strain validation, and unreliable labeling; undermine product reliability and consumer trust. In regions such as the EU and US, probiotics are variably classified as food supplements, dietary supplements, or drugs, complicating regulatory oversight. Insufficient guidelines on strain identification, viable cell counts, and substantiation of health claims often lead to discrepancies between labeled and actual product content. These inconsistencies erode consumer confidence and pose potential public health risks, underscoring the urgent need for harmonized, evidence-based regulatory standards.

Given the widespread co-administration of probiotics with antibiotics in the clinical management of acute and antibiotic-associated diarrhea, evaluating antibiotic susceptibility in probiotic organisms is essential to reflect real-world exposure conditions. Accordingly, this study selected antibiotics representing major classes commonly prescribed for gastrointestinal and systemic infections in India, not to establish therapeutic breakpoints, but to distinguish intrinsic from acquired resistance traits relevant to probiotic survival, persistence, and safety during concomitant antibiotic therapy.

Despite the high burden of acute diarrhea in India and the expanding use of commercial probiotics, systematic data on product quality, strain fidelity, and safety remain limited. To address this gap, we evaluated *S. boulardii*–based probiotic formulations using complementary *in vitro* approaches, including MIC determination, time-kill kinetics, and gastrointestinal simulation. To this, whole-genome sequencing to confirm strain identity and assess antimicrobial resistance determinants, virulence-associated genes, and safety attributes. This integrated framework enables a structured assessment of commercial probiotic performance and directly informs the need for strengthened quality control and harmonized regulatory oversight.

## Methodology

### Enumeration of probiotic

The 13 commercial probiotic products evaluated in this study were purposively selected based on their high prescription frequency, over-the-counter availability, and routine clinical use in managing gastrointestinal conditions in India. The selected products are widely marketed pharmaceutical probiotic formulations listed in recognized Indian drug compendia (e.g., CIMS India and Drug Today) and distributed through pharmacy networks, supporting their relevance to routine clinical practice in India. Also, these products represent globally established probiotic categories, including lactic acid bacteria (notably *Lactobacillus rhamnosus* GG and other *Lactobacillus spp*.), *Bifidobacterium spp*., spore-forming *Bacillus spp*., and yeast-based probiotics (*S. boulardii*). Collectively, these taxa account for the majority of probiotic strains evaluated in clinical trials worldwide and have well-documented roles in gastrointestinal health and diarrheal disease management.^10-12^

Commercial probiotic products were reconstituted as per manufacturer instructions, serially diluted in sterile phosphate-buffered saline (PBS), and plated on appropriate media. *Lactobacillus spp.* was cultured on de Man, Rogosa, and Sharpe (MRS) agar under anaerobic conditions at 37 °C for 48 h.^13^^,^^14^ Bacillus spp. was grown on Brain Heart Infusion Agar (BHIA) anaerobically at 37 °C for 24 h. *S. boulardii* was cultured on Sabouraud Dextrose Agar (SDA) at 30 °C for 48 h. *Enterococcus* spp. were grown on Bile Esculin Agar at 37 °C under anaerobic conditions for 24–48 h, and *Clostridium butyricum* was cultured on Anaerobic Columbia Blood Agar at 37 °C for 48 h.^13^^,^^15^ Colony-forming units (CFUs) were recorded post-incubation. Strain identification was initially performed using MALDI-TOF MS (VITEK MS, bioMérieux) and confirmed by standard biochemical assays. Control strains ensured quality control, and all experiments were conducted in triplicate to ensure reproducibility. The CFU values were calculated using the formula: CFU/g = (average colony count × dilution factor)/volume plated (mL).

For multi-organism probiotic formulations, species-specific or selective media were used for organism-wise CFU enumeration where feasible. When reliable differentiation was not possible (e.g., *Lactobacillus sporogenes*), CFU enumeration was not performed, and organism presence was instead confirmed using MALDI-TOF MS.

### *In vitro* antibiotics susceptibility testing

Minimum Inhibitory Concentration (MIC) testing for probiotic strains was performed using the broth microdilution method in accordance with CLSI and EUCAST guidelines using cation-adjusted Mueller–Hinton broth (CAMHB). Probiotics were cultured on appropriate selective media, and single colonies were inoculated into selective broth and incubated at 37 °C for 18–24 h. Bacterial suspensions were adjusted to 0.5 McFarland standard (~1.5 × 10⁸ CFU/mL).^6^

Antibiotics tested included cefuroxime, cefixime, amoxicillin, amoxicillin/clavulanate, azithromycin, and ciprofloxacin. Serial two-fold dilutions (up to 256 µg/mL) were prepared in 96-well plates. Each well received 100 µL of antibiotic solution and 100 µL of inoculum (final ~5 × 10⁵ CFU/mL). Plates were incubated at 37 °C for 18–24 h, and MICs were visually determined. Reference strains *E. coli* ATCC 25922, *P. aeruginosa* ATCC 27853, and *S. aureus* ATCC 29213 were used for quality control. MIC values were interpreted according to CLSI or EUCAST guidelines, consistent with EFSA recommendations^16^ for standardized MIC determination in antimicrobial susceptibility testing.^17^^,^^18^ For antibiotics not included in EFSA guidance, interpretation was informed by CLSI/EUCAST standards and published probiotic susceptibility studies, were utilized.^6^ Further, the genetic basis of resistance was further assessed by screening for antimicrobial resistance genes using ResFinder (v4.0). All MIC assays were performed in technical duplicates, and quality control strains were included to ensure reproducibility.

### Time-kill

The time-kill assay was conducted to assess the bactericidal activity of selected antibiotics against representative probiotic strains. *Bacillus* spp. and *E. faecalis* were tested in Cation-Adjusted Mueller–Hinton Broth (CAMHB), *Lactobacillus* spp. in de Man, Rogosa, and Sharpe (MRS) broth, and *S. boulardii* in Sabouraud Dextrose Broth. Each assay was initiated with an inoculum of approximately 5 × 10⁵ CFU/mL. Antibiotics tested included cefuroxime, cefixime, amoxicillin, amoxicillin/clavulanic acid, ciprofloxacin, and azithromycin, each at a concentration equivalent to 1× the MIC.

For Bacillus spp. and *E. faecalis*, samples were collected at 0, 2, 4, 6, 8, and 24 hours. For *Lactobacillus spp*. and *S. boulardii*, samples were collected at 0, 3, 6, 9, 24, and 48 hours to accommodate their slower growth kinetics. Viable counts were determined by plating serial dilutions on appropriate selective agar, followed by incubation at 37 °C for 18–24 hours. Colony-forming units per milliliter (CFU/mL) were recorded at each time point.

Bactericidal activity was defined as a ≥3 log₁₀ CFU/mL reduction in viable counts relative to the initial inoculum. All experiments were performed in duplicate, and untreated controls were included to ensure data reliability and accuracy. Results were analyzed to determine the temporal impact of each antibiotic on the survival of the probiotic strains.

### Survival of probiotics under simulated gastrointestinal conditions

The gastrointestinal tolerance of probiotics was evaluated using a stepwise *in vitro* digestion model that simulates the sequential conditions of the mouth, stomach, duodenum, and ileum.^19^ One gram of each probiotic product (powder or capsule content) was suspended in 9 mL of peptone water.

In the oral phase, the pH was adjusted to 6.9 using 0.1 M HCl or 0.1 M NaHCO₃ and incubated at 37 °C with agitation at 200 rpm for 2 minutes. The gastric phase involved the addition of pepsin (25 mg/mL) with stepwise pH reductions (5.5, 4.6, 3.8, 2.8, 2.3, and 2.0) over 90 minutes at 37 °C with 130 rpm shaking. After this phase, aliquots were collected, serially diluted, and plated on selective agar to assess viability.

For the duodenal phase, pancreatin (2 g/L) and bovine bile salts (12 g/L) were added to a final concentration of 0.25 mL/mL, with the pH adjusted to 5.0. The mixture was incubated at 37 °C with 45 rpm agitation for 20 minutes, followed by sampling and plating. In the ileum phase, the pH was raised to 6.5 using NaHCO₃ and incubated at 37 °C with 90 rpm agitation for 90 minutes. A final aliquot was collected, diluted, and plated.

All plates were incubated anaerobically at 37 °C for 48 hours. Survivors were quantified and expressed as log₁₀ CFU/g. This method enabled the assessment of probiotic viability and resilience throughout simulated gastrointestinal transit.

### Genomic DNA extraction and sequencing

Genomic DNA was extracted using the PowerSoil DNA Isolation Kit (Qiagen, USA). The quality and quantity of the extracted DNA were assessed using a NanoDrop spectrophotometer. DNA concentrations suitable for sequencing were confirmed, and library preparation was carried out using the Nextera DNA Flex Library Prep Kit (Illumina, San Diego, CA, USA). The final libraries were sequenced on an Illumina HiSeq platform, generating high-quality sequencing reads. Adapter sequences were removed using Cutadapt v1.8.1, and read quality was assessed using FastQC v0.11.4. Reads with a PHRED quality score below 20 were discarded to ensure high-quality data for downstream analysis.

### Bioinformatic analysis

The genomic analysis of probiotic strains was performed using a comprehensive bioinformatics tool to assess genome assembly, annotation, and functional characterization. Genome assembly was conducted using Unicycler v0.4.8, generating a high-quality draft assembly. Assembly quality was evaluated using CheckM v1.0.5 and QUAST v4.5 to determine genome completeness, contamination, misassemblies, mismatches, and indels. Genome annotation was performed using Prokka v1.14.5 (https://github.com/tseemann/prokka), facilitating gene prediction and functional annotation. Plasmid types were identified using Plasmidfinder (v2.1, https://cge.food.dtu.dk/services/PlasmidFinder/). Acquired antimicrobial resistance genes (ARGs) and chromosomal mutations associated with resistance were identified using ResFinder (v4.0, https://cge.food.dtu.dk/services/ResFinder/). Virulence factors were detected using the VFanalyzer in the Virulence Factor Database as well as VirulenceFinder (https://cge.food.dtu.dk/services/VirulenceFinder/). Although WGS-based analytical pipelines are well documented, there is no unified framework tailored for concurrent analysis of highly diverse bacterial species. Accordingly, species-appropriate tools and reference databases were used for genomic identification and safety screening in this study. This heterogeneity limits direct cross-species comparability of genomic features and represents an inherent constraint when applying in silico analyses across mixed probiotic taxa.

## Results

### Discrepancies in CFU counts and taxonomy of commercial probiotics

Thirteen commercially available probiotic products were evaluated using colony-forming unit (CFU) enumeration and matrix-assisted laser desorption/ionization–time of flight (MALDI-TOF) mass spectrometry to assess the accuracy of labeled microbial content. Discrepancies between the declared and observed CFU counts were identified in five products: Bifilac Sachet, Nutrolin B, Enterogermina (Vial), Sporit GG (against *S. boulardii*), and Ecoviva. This observation indicates potential issues with product viability and stability. Although the majority of products exhibited concordance between the labeled strains and those identified via MALDI-TOF analysis, inconsistencies were detected in certain formulations. In particular, Bifilac Sachet, labeled as containing *Streptococcus faecalis*, was identified as *Enterococcus faecalis* and *Enterococcus faecium*, suggesting both taxonomic misclassification and possible microbial contamination. Additionally, outdated or non-standard bacterial nomenclature was observed on several product labels. For example, Sporlac Powder and Bifilac Sachet listed *L. sporogenes*, a nomenclature that has been taxonomically reclassified as *B. coagulans*. Similarly, Nutrolin B was labeled as containing “Lactic acid bacillus,” a term lacking taxonomic validity, while MALDI-TOF analysis confirmed the presence of *B. coagulans*, a spore-forming, lactic acid-producing bacterium. A detailed summary of the findings is provided in Table 1.

**Table 1.** Enumeration and MALDI-TOF-based identification of commercial probiotic products.

S. no.	Brand (company)	Labeled probiotic(s)	MALDI-TOF identification	Labeled CFU	Enumerated CFU	Remarks
1	Sporlac powder (Sanzyme Pvt. Ltd.)	*Lactobacillus sporogenes*	*Bacillus coagulans*	1.5 × 10⁸	1 × 10⁸	CFU matched; organism matched
2	Bifilac Sachet (Tablets (India) Ltd.)	*Bacillus mesentericus* TO-A	*Bacillus subtilis*	1 × 10^6^	3 × 10⁷	CFU matched; organism matched
		*Streptococcus faecalis* T-110	*Enterococcus faecalis*	3 × 10⁷	2 × 10⁸	CFU matched; organism matched; ***E. faecium*** **contamination detected**
		*Clostridium butyricum* TO-A	*Clostridium butyricum*	2 × 10⁶	5 × 10⁷	CFU matched; organism matched
		*Lactobacillus sporogenes*	*Bacillus coagulans*	5 × 10⁷	Not determined	CFU not determinable; organism matched
3	Sporlac GG (Allianz Biosciences Pvt. Ltd.)	*Lactobacillus rhamnosus* GG ATCC 53103	*Lactobacillus rhamnosus*	6 × 10⁹	8 × 10⁹	CFU matched; organism matched
4	Tufpro (Sun Pharma)	*Bacillus clausii* UBBC-07	*Bacillus clausii*	2 × 10⁹	1 × 10⁹	CFU matched; organism matched
5	Nutrolin B Capsule (Cipla)	Lactic Acid Bacillus	*Bacillus coagulans*	4 × 10⁷	6 × 10⁷	CFU matched; organism matched
6	Enterogermina Capsule (Sanofi India Ltd.)	*Bacillus clausii* O/C, N/R, SIN, T	*Bacillus clausii*	2 × 10⁹	6 × 10⁷	**CFU not matched**; organism matched
7	Darolac Syrup (Aristo Pharmaceuticals)	*Bacillus subtilis* HU58	*Bacillus subtilis*	2 × 10⁹	4 × 10⁹	CFU matched; organism matched
8	GNorm (Nouveau Medicament)	*Saccharomyces boulardii*	*Saccharomyces cerevisiae*	1 × 10⁹	6 × 10⁹	CFU matched; organism matched
9	Enterogermina Vial. (Sanofi India Ltd)	*Bacillus clausii* O/C, N/R, SIN, T	*Bacillus clausii*	2 × 10⁹	5 × 10⁸	**CFU not matched**; organism matched
10	Nutrolin B Syrup (Cipla)	Lactic Acid Bacillus	*Bacillus coagulans*	4 × 10^7^	3 × 10⁶	**CFU not matched**; organism matched
11	Sporit GG (Sun Pharma)	*Saccharomyces boulardii* CNCM I-3799	*S. cerevisiae*	5 × 10⁹	1 × 10⁸	**CFU not matched**; organism matched
		*L. rhamnosus* GG ATCC 53103	*L. rhamnosus*	5 × 10⁹	2 × 10⁹	CFU matched; organisms matched
12	Econorm (Biocodex)	*Saccharomyces boulardii* CNCM I-745	*Saccharomyces cerevisiae*	5 × 10⁹	3 × 10⁹	CFU matched; organism matched
13	Ecoviva (Granules (India) Ltd.)	*Saccharomyces boulardii* CNCM I-3799	*Saccharomyces cerevisiae*	5 × 10⁹	3 × 10⁸	**CFU not matched**; organism matched

Note: The table summarizes the manufacturer-declared probiotic composition and viable counts alongside experimentally enumerated CFU values and species-level identification using MALDI-TOF MS. Labeled and enumerated CFU values correspond to the unit specified by the manufacturer (e.g., per g, per 0.5 g, per capsule, per 5 mL, or per mL). Agreement between labeled and enumerated CFU counts was interpreted using a log-scale tolerance commonly applied in microbiological enumeration, where deviations within ±0.5–1.0 log₁₀ CFU are generally considered acceptable due to inherent analytical and biological variability. Enumeration data are presented descriptively to assess concordance with label claims; formal statistical comparisons between products were not performed. Bold values indicate contamination, organism mismatch, or major CFU discrepancies.

### *In vitro* susceptibility testing

**Figure 1.**
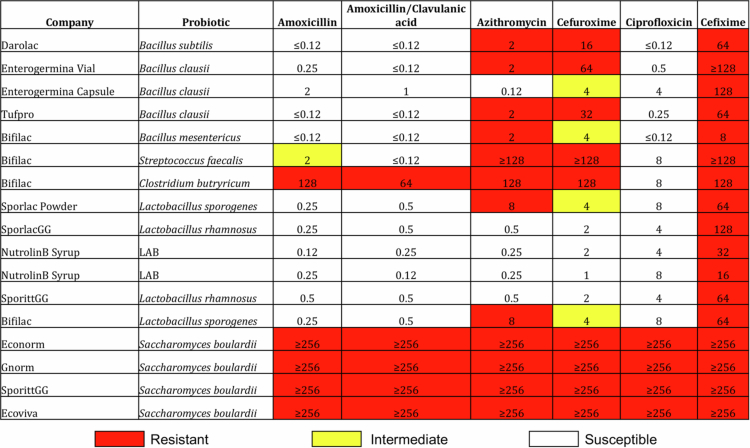
*In vitro* antibiotic susceptibility profile of commercial probiotic products. The table summarizes the MICs of various antibiotics against commercially available probiotic strains. MICs were interpreted based on standard breakpoints, with red, yellow, and white indicating resistant, intermediate, and susceptible categories, respectively.

*In vitro* susceptibility testing of probiotic strains retrieved from the commercial probiotics products was done against six clinically relevant antibiotics, revealing diverse resistance patterns, with minimum inhibitory concentrations (MICs) ranging from 0.06 to 256 mg/L (Figure 1). Notably, all tested strains demonstrated resistance to cefixime, with MIC values spanning 8–256 mg/L.

Among *Bacillus* spp., most isolates exhibited susceptibility to amoxicillin, amoxicillin/clavulanate, and ciprofloxacin. However, *Bacillus clausii* (isolated from Enterogermina) displayed intermediate resistance to amoxicillin (MIC: 2 mg/L) and ciprofloxacin (MIC: 4 mg/L). Additional *Bacillus* isolates, including *Bacillus mesentericus* from Bifilac, exhibited resistance to cefixime, azithromycin, and cefuroxime, though with relatively lower MICs compared to other resistant strains. Interestingly, although both Enterogermina formulations (vial and capsule) contain the same four *Bacillus clausii* strains, the vial formulation showed resistance to azithromycin and cefuroxime, consistent with the WGS analysis. For *Lactobacillus* spp., all isolates were susceptible to amoxicillin and amoxicillin/clavulanate. Some strains exhibited intermediate resistance to cefuroxime (MIC: 4 mg/L). Notably, *L. sporogenes* from Sporlac Powder showed reduced susceptibility to azithromycin, with MICs reaching up to 8 mg/L.

The *E. faecalis* isolate from Bifilac demonstrated high-level resistance to azithromycin and cefuroxime (MIC ≥ 128 mg/L), while remaining susceptible to amoxicillin and amoxicillin/clavulanate (MIC ≤ 0.12 mg/L). This strain also exhibited resistance to ciprofloxacin (MIC: 8 mg/L).

*C. butyricum* (Bifilac) displayed extensive resistance, with MIC values ≥128 mg/L for azithromycin, cefuroxime, cefixime, and amoxicillin. This strain also showed reduced susceptibility to amoxicillin/clavulanate (MIC: 64 mg/L) and moderate resistance to ciprofloxacin (MIC: 8 mg/L). Among the bacterial probiotics, variation in resistance patterns observed among the same organisms from different commercial products indicates strain-specific differences in antibiotic susceptibility.

As anticipated based on its fungal taxonomy, *S. boulardii* (present in Econorm and Sporlac CG) exhibited intrinsic resistance to all tested antibiotics, with MICs ≥ 256 mg/L.

### Time-kill assay

A time-kill assay on eight representative probiotic strains, each derived from a distinct species, was conducted to evaluate viability under clinically relevant antibiotic exposure. For bacterial strains, antibiotics were applied at their respective minimum inhibitory concentrations (MICs), whereas *S. boulardii*, known to exhibit intrinsic resistance to all tested antibiotics, was exposed to the maximum serum concentration (*C*_max_) of each agent.

**Figure 2.**
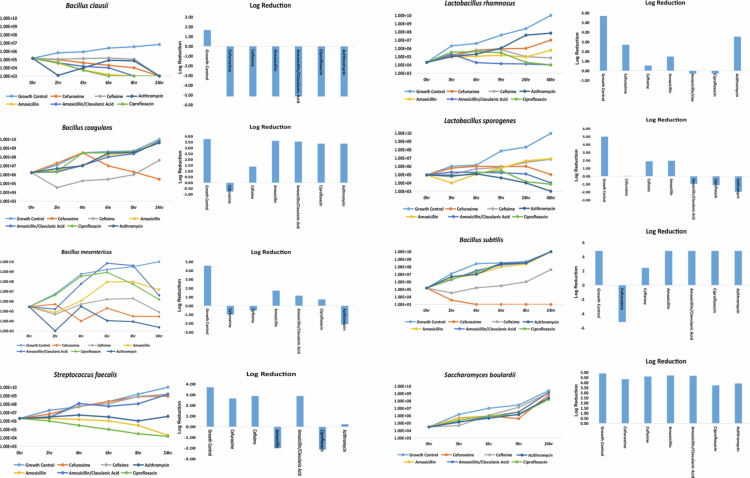
Time-kill assay of commercial probiotic strains against selected antibiotics. This figure illustrates the time-kill kinetics (left panels) and corresponding log₁₀ CFU/mL reduction (right panels) of various probiotic formulations upon exposure to antibiotics. Bacterial probiotics were tested at 1× MIC concentrations, while *Saccharomyces boulardii* was tested at Cmax levels. Each graph shows bacterial/fungal viability over time (0–24 or 48 hours), with antibiotics: cefuroxime, cefixime, amoxicillin, amoxicillin–clavulanate, ciprofloxacin, and azithromycin. The bar graphs depict log₁₀ reduction relative to the initial inoculum.

As depicted in Figure 2, all bacterial strains displayed a significant logarithmic reduction in viable counts, revealing time-dependent bactericidal effects not fully captured by static MIC assays. Particularly, *Bacillus* spp., despite demonstrating azithromycin resistance in standard MIC assays, showed substantial killing in the time-kill assay, highlighting the ability of dynamic assays to uncover latent susceptibilities. In contrast, *S. boulardii* exhibited no notable growth inhibition.

### *In vitro* gastrointestinal (GI) simulation assay

*In vitro* gastrointestinal (GI) simulation assay was done to assess the survival of 13 probiotic formulations across simulated GI compartments, including the stomach, duodenum, and ileum. Viability was quantified as log colony-forming units per milliliter (log CFU/mL) and expressed as mean ± standard deviation (SD) at each stage, starting from the initial inoculum (Table 2).

**Table 2.** Survival of commercial probiotics during simulated gastrointestinal transit.

Probiotic	Initial	Stomach	Duodenum	Ileum	Δlog₁₀	Remark
Econorm	9.48 ± 0.8	8.62 ± 0.2	9.15 ± 0.5	9.7 ± 0.1	+0.22	Growth/enrichment during transit
Ecoviva	8.48 ± 0.5	7.45 ± 0.2	7.46 ± 0.1	8.61 ± 0.5	+0.13	Growth/enrichment during transit
Sporlac GG	9.67 ± 0.2	7.32 ± 0.6	6.98 ± 0.8	6.48 ± 0	−3.19	Severe loss (>1000-fold)
Sporit GG	8 ± 0	6.43 ± 0.2	6.33 ± 0.1	6.31 ± 0.3	−1.69	Moderate–severe loss
Sporlac powder	8 ± 0	7.41 ± 0.2	7.26 ± 0.3	8.07 ± 0.1	+0.07	Stable survival
Nutrolin B	6.64 ± 0.8	7.57 ± 0.2	6.49 ± 0.1	6.67 ± 0.2	+0.03	Growth/enrichment during transit
Nutrolin B Syrup	6.48 ± 0.5	7.15 ± 0.1	7.45 ± 0.3	7.3 ± 0	+0.82	Growth/enrichment during transit
Bifilac	8.45 ± 0.4	7.49 ± 0.5	8.33 ± 0.3	8.43 ± 0.4	−0.02	Stable survival
Darolac	9.6 ± 0.1	7.62 ± 0.4	7.07 ± 0.4	7.23 ± 0.1	−2.37	Severe loss
Tufpro	9 ± 0	6.59 ± 0.5	8.48 ± 0.1	6.74 ± 0.1	−2.26	Severe loss
Enterogermina (Vial)	8.7 ± 0.5	6.61 ± 0.1	6.83 ± 0.6	6.44 ± 0.1	−2.26	Severe loss
Entergermina (capsule)	9.6 ± 0.1	6.62 ± 0.5	7.43 ± 0.3	6.51 ± 0.1	−3.09	Severe loss
Gnorm	9.6 ± 0.6	6.92 ± 0.1	6.99 ± 0.6	7.05 ± 0.1	−2.55	Severe loss

Note: Changes in viable counts across gastrointestinal simulation stages were expressed as mean log₁₀ CFU ± SD. Variations within ±0.5–1.0 log₁₀ CFU were considered acceptable, reflecting the inherent analytical variability of plate count–based enumeration and biological stress responses during *in vitro* gastrointestinal simulation. Log retention was calculated as Δlog₁₀ = log₁₀(CFU_ileum) − log₁₀(CFU_initial) and interpreted as good retention (Δlog₁₀ ≥ −1; ≤10-fold loss), moderate retention (Δlog₁₀ −1 to −2), and poor retention (Δlog₁₀ < −2; >100-fold loss).

Initial viable counts ranged from 6.48 ± 0.5 log CFU/mL (Nutrolin B Syrup) to 9.67 ± 0.2 log CFU/mL (Sporlac CG). Following exposure to simulated gastric conditions, all probiotics exhibited a reduction in viability, with counts decreasing to 6.59 ± 0.5 log CFU/mL (Tufpro) to 8.62 ± 0.2 log CFU/mL (Econorm). In the duodenal phase, viable counts ranged from 6.33 ± 0.1 log CFU/mL (Sporit GG) to 9.15 ± 0.5 log CFU/mL (Econorm). By the ileal stage, counts stabilized or slightly decreased, ranging from 6.48 ± 0.0 log CFU/mL (Sporlac CG) to 9.7 ± 0.1 log CFU/mL (Econorm).

Econorm, containing *S. boulardii*, demonstrated the highest survival across all GI compartments, with viable counts of 8.62 ± 0.2 log CFU/mL in the stomach, 9.15 ± 0.5 log CFU/mL in the duodenum, and 9.7 ± 0.1 log CFU/mL in the ileum. Sporit GG, a formulation combining *S. boulardii* and *L. rhamnosus*, GG exhibited the lowest duodenal survival (6.33 ± 0.1 log CFU/mL) but showed slight recovery in the ileum (6.31 ± 0.1 log CFU/mL). *Sporlac CG*, despite a high initial count of 9.67 ± 0.2 log CFU/mL, displayed the lowest ileal survival (6.48 ± 0.0 log CFU/mL), indicating pronounced sensitivity to GI conditions. Other probiotics, including Ecoviva, Bifilac, and Darolac, maintained moderate viability, with ileal counts ranging from 7.23 ± 0.0 log CFU/mL (Darolac) to 8.61 ± 0.5 log CFU/mL (Ecoviva).

### Genome-wide sequence analyses of the probiotics

Genome sequencing was performed on probiotic products to confirm the identity of the microorganisms and to assess the presence of resistome and virulome determinants. Whole-genome sequencing results were compared with MALDI-TOF identifications to evaluate concordance between phenotypic and genomic classification and to interpret organism identity using current taxonomic nomenclature (Table 3). The results revealed discrepancies between the product labels and genomic identifications.

**Table 3.** Comparative species identification of probiotic products using label claims, MALDI-TOF MS, and WGS-based methods. Species-level identification of probiotic products based on manufacturer label claims, MALDI-TOF MS, and whole-genome sequencinG (PathogenWatch and rMLST). The interpretation column indicates concordance or discordance across methods and notes outdated or revised taxonomy. NA denotes data not applicable or unavailable.

Product	Label claim	MALDI-TOF ID	PathogenWatch ID	rMLST ID	Interpretation
Sporlac powder	*Lactobacillus sporogenes*	*Bacillus coagulans*	*Bacillus coagulans*	*Heyndrickxia faecalis/H. coagulans*	Label claim outdated; WGS confirms *B. coagulans*
Nutrolin	LAB	*Bacillus coagulans*	*Bacillus coagulans*	*Heyndrickxia faecalis/H. coagulans*	LAB claim nonspecific; correctly identified as *B. coagulans*
Nutrolin B Syrup	LAB	*Bacillus coagulans*	*Bacillus coagulans*	*Heyndrickxia faecalis*	Consistent identification as *B. coagulans*
Bifilac	*Bacillus mesentericus* TO-A	*B. subtilis/B. vallismortis*	*Bacillus subtilis*	*Bacillus subtilis*	Label mismatch; WGS supports *B. subtilis*
Bifilac	*Lactobacillus sporogenes*	*Bacillus coagulans*	*Bacillus subtilis*	*Bacillus subtilis*	Major label–WGS discordance
Darolac	*Bacillus subtilis* HU58	*Bacillus subtilis*	*Bacillus subtilis*	*Bacillus subtilis*	Fully concordant across all methods
Tufpro	*Bacillus clausii* UBBC-07	*Bacillus clausii*	*Bacillus clausii*	*Shouchella rhizosphaerae*	Genus concordant; rMLST reflects recent taxonomic revision
Enterogermina (vial)	*Bacillus clausii* O/C, N/R, SIN, T	*Bacillus clausii*	*Bacillus clausii*	*Shouchella rhizosphaerae*	Genus concordant; rMLST reflects recent taxonomic revision
Enterogermina (capsule)	*Bacillus clausii* O/C, N/R, SIN, T	*Bacillus clausii*	*Bacillus clausii*	*Shouchella rhizosphaerae*	Genus concordant; rMLST reflects recent taxonomic revision
Sporit GG	*Lactobacillus rhamnosus* ATCC 53103	*Lactobacillus rhamnosus*	*Lactobacillus rhamnosus*	*Lactobacillus rhamnosus*	Fully concordant across all methods
Sporlac GG	*Lactobacillus rhamnosus* ATCC 53103	*Lactobacillus rhamnosus*	*Lactobacillus rhamnosus*	*Lactobacillus rhamnosus*	Fully concordant across all methods
Econorm	*Saccharomyces boulardii* CNCM I-745	*Saccharomyces cerevisiae*	*Saccharomyces cerevisiae*	NA	Expected synonymy; *S. boulardii* is a subtype of *S. cerevisiae*

Probiotics labeled as *L. sporogenes* and “LAB (Lactic Acid Bacteria)” were identified through genome sequencing as *Heyndrickxia faecalis*/*Heyndrickxia coagulans* and *Heyndrickxia faecalis*, respectively, with PathogenWatch analysis indicating the presence of *B. coagulans*. Products labeled as *B. mesentericus*, *L. sporogenes*, and *B. subtilis* were all identified as *B. subtilis*. Similarly, products labeled as *B. clausii* were identified by rMLST as *Shouchella rhizosphaerae*; however, PathogenWatch analysis confirmed the presence of *B. clausii*. In contrast, probiotics labeled as *Lactobacillus rhamnosus* were correctly identified as *L. rhamnosus* by both genome sequencing and PathogenWatch analyses.

### Identification of AMR and VF determinants in probiotics

**Figure 3.**
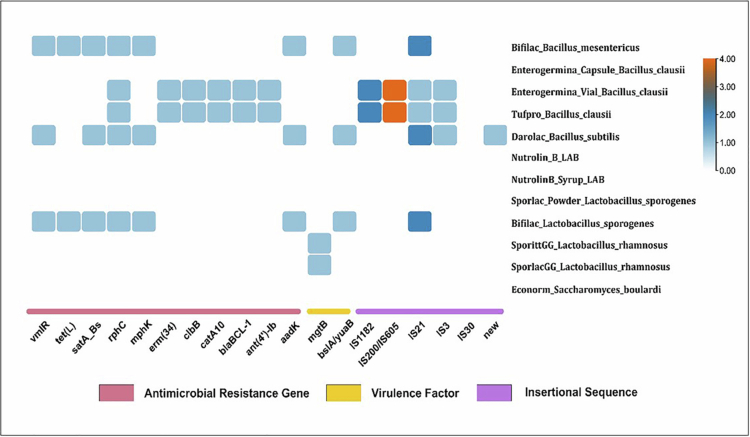
Antimicrobial resistance genes (ARG) and virulence genes for probiotic isolates. The presence or absence of ARG is show in red line; virulence genes are denoted in yellow line and the IS elements are denoted in purple line. The color of the square box represents the copy number of the respective IS elements.

Probiotic safety in this study was assessed using a risk-based microbiological framework incorporating strain-level identification, in silico screening for virulence-associated determinants, evaluation of antimicrobial resistance genes with attention to transferability, and phenotypic susceptibility profiles interpreted in the context of intrinsic resistance. Consistent with established guidance, probiotic safety is linked to intended use, strain identity, and historical evidence of safe exposure, and is distinct from classical pathogenic risk assessment.^20-22^ The antimicrobial resistance (AMR) and virulence factors (VF) in the probiotic samples are shown in Figure 3. Several of the probiotics carried the virulence factor *bslA/yuaB* (biofilm surface layer protein A). Specifically, detected in Bifilac *B. mesentericus*, Bifilac *L. sporogenes*, and Darolac *B. subtilis*. The *mgtB* (magnesium transporter) was identified in the Sporit GG *L. rhamnosus* and Sporlac GG *L. rhamnosus*. The *vmlR* (ABC-F type ribosomal protection protein), *tet(L)* (tetracycline efflux MFS transporter), *satA_Bs* (streptothricin *N*-acetyltransferase), *rphC* (rifamycin-inactivating phosphotransferase), *mphK* (macrolide 2ʹ-phosphotransferase), and *aadK* (aminoglycoside 6-adenylyltransferase) genes were present in all three of *Bifilac B. mesentericus*, *L. sporogenes*, and *Darolac B. subtilis*. Except *tet(L)*in the *Darolac B. subtilis*. The *erm(34)* (23S rRNA (adenine(2058)-N(6))-methyltransferase), *clbB* (23S rRNA (adenine(2503)-C(8))-methyltransferase), *catA10* (type A-10 chloramphenicol O-acetyltransferase), *blaBCL-1* (class A beta-lactamase), and *ant(4ʹ)-lb* (aminoglycoside O-nucleotidyltransferase) genes were present in the *Enterogermina Vial B. clausii* and *Tufpro B. clausii*. In contrast, Econorm *S. boulardii*, Nutrolin *B LAB*, and Nutrolin B Syrup LAB showed no evidence of carrying any of the tested AMR genes or VFs. These observations are illustrated in Figure 3.

## Discussion

This comprehensive evaluation of 13 commercial probiotic products marketed in India reveals substantial challenges regarding quality, labeling accuracy, and safety, which may significantly impact clinical outcomes, particularly in the management of acute diarrhea. Despite the global recognition of probiotics as therapeutic adjuncts, our findings underscore the urgency of improved regulatory oversight and standardized characterization to ensure their safe and effective use.

A significant number of tested products (5 of 13) failed to meet their labeled colony-forming unit (CFU) claims, as determined through laboratory enumeration. Similar studies conducted elsewhere have shown products such as *β-LOCK®*, *BENEGUT®*, and *PROALANA-B®* contain substantially fewer viable organisms than declared, potentially compromising therapeutic efficacy. Conversely, *ENTEROGERMINA®* and *TUFPRO®* matched their label claims, suggesting variability in manufacturing practices.^23^ These findings align with global assessments, which report label-claim discrepancies in over 40% of commercial probiotics.^24^^,^^25^

A recurring issue across multiple products was the discrepancy between labeled and actual contents, as revealed by MALDI-TOF-based identification and genomic sequencing. Five of the thirteen products, including Bifilac Sachet, Nutrolin B, and Enterogermina—demonstrated labeling inaccuracies or contamination. For instance, Bifilac Sachet was labeled as containing *Streptococcus faecalis*, a deprecated term, but was found to contain *E. faecalis* and *E. faecium*, indicating either taxonomic confusion or potential contamination.^26^ Similarly, Nutrolin B and Sporlac Powder used the outdated and ambiguous designation “Lactic Acid Bacillus” or “*Lactobacillus sporogenes*,” both of which are misnomers for *B. coagulans*.

Such generic or outdated nomenclature hinders accurate assessment of probiotic efficacy, as health benefits are inherently strain-specific. Products failing to specify strain-level identity prevent clinicians and consumers from making informed choices and raise concerns over misidentification-related safety issues, particularly in vulnerable populations.^27^ These findings highlight the need for stricter regulation, better quality control, and adherence to updated taxonomy to ensure accurate probiotic labeling.

*In vitro* susceptibility revealed notable strain-specific antibiotic resistance among commercial probiotics, even within the same species. While most *Bacillus* and *Lactobacillus* strains were susceptible to amoxicillin, strains like *B. clausii*, *B. mesentericus*, *C. butyricum*, and *E. faecalis* showed multidrug resistance. Universal cefixime resistance and inter-product variability among bacterial probiotics highlight the need for strain-specific susceptibility testing.^28^

These findings demonstrate that antibiotic resistance is highly strain-dependent, challenging the reliability of species-level assumptions. To safeguard public health, regulatory bodies should require strain-specific resistance profiling for probiotics. This is crucial not only to prevent resistance gene transfer but also to ensure that probiotics do not compromise antibiotic therapy. The observed resistance raises two key concerns: diminished effectiveness when probiotics are used alongside antibiotics, and their potential role in exacerbating antimicrobial resistance (AMR) within the gut microbiome.

Notably, *S. boulardii* (Econorm) demonstrated intrinsic resistance to all tested antibiotics due to its eukaryotic nature, a characteristic that supports its use during antibiotic therapy without compromising viability.

Time-kill assays confirmed the vulnerability of bacterial probiotics in antibiotic-rich environments, even among those exhibiting apparent resistance in static tests. In contrast, *S. boulardii* maintained viability under peak serum antibiotic concentrations, underscoring its clinical utility during treatments that disrupt gut flora, such as antibiotic-associated diarrhea (AAD).

The *in vitro* gastrointestinal (GI) simulation assay demonstrated significant variability in the survival of 13 probiotic formulations across simulated GI compartments, highlighting strain- and formulation-specific resilience. Initial viable counts ranged broadly, with Sporlac CG showing the highest starting concentration (9.67 ± 0.2 log CFU/mL) and Nutrolin B Syrup the lowest (6.48 ± 0.5 log CFU/mL). All products experienced viability loss in the stomach, where acidic conditions and pepsin are most hostile. However, Econorm, containing *S. boulardii* CNCM I-745, displayed exceptional gastric survival (8.62 ± 0.2 log CFU/mL) and maintained the highest counts throughout all stages, including the duodenum (9.15 ± 0.5) and ileum (9.7 ± 0.1), indicating strong resilience. In contrast, formulations like Sporit GG and Sporlac CG, despite initially high counts, showed pronounced sensitivity to GI conditions, especially in the duodenum and ileum, respectively.

Probiotic effectiveness relies not just on initial viable counts but also on the strain's ability to survive digestive stresses. *S. boulardii* showed strong resilience, making it suitable for oral use where high survivability is crucial. In contrast, some multi-strain or bacterial-only products showed reduced viability, likely due to poor formulation or negative strain interactions. These results highlight the importance of GI simulation models for evaluating and optimizing probiotic formulations before clinical application, ensuring effective delivery of live organisms to the intestine.

WGS analysis revealed multiple antimicrobial resistance genes (ARGs), virulence factors (VFs), and insertion sequence (IS) elements across several probiotic isolates (Figure 3; Supplementary Table 1), informing their potential for horizontal gene transfer (HGT). Mobility was inferred based on gene localization, association with mobile genetic elements, and the presence of insertion sequences. Genes located on plasmids or transposons were classified as high mobility, those occasionally linked to mobile elements as moderate, and chromosomally encoded intrinsic genes as low mobility, providing a framework to assess dissemination risk.

For example, Bifilac isolates (*Bacillus mesentericus* and *Bacillus coagulans*) and Darolac (*Bacillus subtilis*) harbored resistance genes including *vmlR, tet(L), satA_Bs, rphC, mphK,* and *aadK*, representing mechanisms such as ribosomal protection, efflux, and enzymatic antibiotic modification. Based on mobility classification criteria, *tet(L)* showed higher predicted mobility, *mphK* and *aadK* exhibited moderate mobility, whereas *vmlR, satA_Bs,* and *rphC* were considered low-mobility intrinsic determinants.

Similarly, Enterogermina and Tufpro isolates (*Bacillus clausii*) contained *erm(34), clbB, catA10, blaBCL-1,* and *ant(4′)-Ib*. Among these, *catA10* and *ant(4′)-Ib* demonstrated moderate–high predicted mobilizability, whereas *erm(34), blaBCL-1, and clbB* are generally regarded as chromosomally encoded intrinsic resistance determinants with low mobility. Virulence-associated genes were also detected in specific isolates, including *hngB* in Bifilac *(B. mesentericus)* and *bslA/yugB* in Darolac *(B. subtilis).* In addition, several insertion sequence elements (IS1182, IS200/IS605 family, IS21, IS3, IS30, and a putative novel IS element) were identified, suggesting the presence of mobile genetic elements that may contribute to genomic plasticity.

The presence of such ARGs and VFs may have clinical relevance, particularly in immunocompromised individuals or those with disrupted gut barriers, where opportunities for opportunistic infection or horizontal gene transfer may be increased.^22^^,^^29-35^ Genomic studies of the composite genome of *Bacillus clausii* from the probiotic Enterogermina® formulation have similarly reported a mobilome enriched with transposons and other mobile genetic elements, along with a novel plasmid sequence.^36^ Consistent with these observations, *B. clausii* isolates in this study showed a comparatively higher abundance of antimicrobial resistance genes and IS elements. However, many resistance determinants identified in *Bacillus* probiotics are considered intrinsic chromosomal traits, and therefore, their presence alone does not necessarily indicate horizontal gene transfer without further analysis of their genomic context.^8^^,^^28^

These findings highlight the limitations of species- or label-based safety assumptions and underscore the necessity of strain-specific assessment. Conversely, Econorm exhibited no ARGs or virulence determinants, reinforcing its favorable safety profile and supporting its recommendation for use in immunocompromised populations where bacterial probiotics may pose infection risks.

Among the tested products, Econorm, containing *S. boulardii* CNCM I-745, emerged as the most reliable and clinically suitable probiotic for acute diarrhea. Its advantages are multifaceted: accurate strain identification, superior GI survivability, intrinsic antibiotic resistance without transferable genes, and a clean safety profile. In contrast to bacterial probiotics, *S. boulardii*–based products lacked detectable ARGs or virulence factors while exhibiting intrinsic, non-transferable antibiotic resistance, supporting a comparatively favorable safety profile in high-risk clinical settings.^4^^,^^9^^,^^36^^,^^37^ Clinical studies have previously established *S. boulardii*'s efficacy in reducing diarrhea duration in pediatric and adult populations, particularly in the context of AAD, *Helicobacter pylori*, and *Clostridioides difficile* infections.^9^^,^^36^^,^^37^

Globally, regulatory inconsistency prevails.^38^ The European Union lacks dedicated probiotic legislation, though health claims are regulated. Japan, Brazil, and Canada offer more structured frameworks that mandate safety and efficacy evidence.^39^ Given the projected growth of India's probiotic market to US$961.856 million by 2025, regulatory harmonization with global best practices is both urgent and feasible.^40^ From a regulatory perspective, our findings support pragmatic, risk-proportionate measures, including mandatory strain-level genomic validation at product approval and selective postmarket genomic surveillance of widely used or higher-risk formulations.

In India, the current regulatory framework, primarily overseen by the Food Safety and Standards Authority of India,^41^ treats probiotics as health supplements under the 2016 regulations. While the ICMR-DBT guidelines encourage strain-level identification and safety testing, there remains no pre-market approval requirement, leaving quality largely at the discretion of manufacturers (https://main.icmr.nic.in/sites/default/files/guidelines/Probiotics_Guidelines.pdf). This absence of stringent oversight allows generic labeling, misidentification, and underdosing to persist, endangering consumer safety.^42^^,^^43^ Targeted batch-wise testing focusing on CFU concordance and key ARGs/VFs could provide a feasible regulatory checkpoint within existing frameworks.

The marketing of probiotics as FSSAI-certified supplements often lacks clinical and genomic validation, raising safety concerns (Table 4). Products like Tufpro, Ecogro, and Progermila bambini have contained opportunistic pathogens such as *Bacillus cereus*, *Acinetobacter* spp., and *Pseudomonas* spp.^30^ Cases of fungemia and fatal mucormycosis linked to *S. boulardii* and *Rhizopus oryzae* highlight risks for vulnerable populations.^32^

**Table 4.** Regulatory status, strain information, cautions, and clinical evidence for selected commercial probiotic brands: Summary of declared organisms, available strain information, regulatory classification, licensing status, usage cautions, and representative clinical evidence for selected commercial probiotic brands based on product labels and publicly available sources.

Probiotic brand	Declared organism(s)	Strain(s)	Product category	Certified by/license	Caution/directions for use	RCT reference(s)
Econorm	*Saccharomyces boulardii*	CNCM I-745	Therapeutic use	Import License No: IL/FF-000214 (FF 615)	To be sold only on the prescription of a registered medical practitioner	[35]
Ecoviva	*Saccharomyces boulardii*	CNCM I-3799	Health supplement & Probiotic food	FSSAI	To be given under medical advice only	[43]
Enterogermina	*Bacillus clausii*	O/C, N/R, SIN, T	Therapeutic use	Import License No: FF-655-24038	No caution specified	[54]
Nutrolin B (syrup)	Lactic acid Bacillus	Not specified	Food health supplement	FSSAI	No caution specified	Strain not mentioned
New Nutrolin B (capsule)	Lactic acid Bacillus	Not specified	Therapeutic use	M.L: 31/UA/SC/P-2018	No caution specified	Strain not mentioned
Sporit GG	*Saccharomyces boulardii*	CNCM I-3799	Probiotic food	FSSAI	Consult a healthcare professional if pregnant, lactating, or on medication	[43]
	*Lactobacillus rhamnosus* GG	ATCC 53103		FSSAI	Same as above	[45]
Sporlac GG	*Lactobacillus rhamnosus* GG	ATCC 53103	Probiotic food	FSSAI	To be given only under medical advice	[45]
Sporlac (powder)	*Lactobacillus sporogenes*	Not specified	Not specified	Mfg. License No: G/28A/6998-A 25/UA/LL/SC/P-2022	As directed by the physician	Strain not mentioned
Darolac	*Bacillus subtilis*	HU58	Probiotic food	FSSAI	Medical advice required for children (2–5 y), pregnant/lactating women, and those with comorbidities	[46]
Tufpro	*Bacillus clausii* spores	UBBC-07	Probiotic food	FSSAI	To be given only under medical advice	[44]
Gnorm	*Saccharomyces cerevisiae* subsp. *boulardii*	Not specified	Probiotic food	FSSAI	Probiotic food; not for medical use	Strain not mentioned
Bifilac	*Streptococcus faecalis*	T-110	Pre- & probiotic	Mfg. License No: 04 23 1117	To be sold only on the prescription of a registered medical practitioner	[47]
	*Bacillus mesentericus*	TO-A				[47]
	*Clostridium butyricum*	TO-A				[47]
	Lactic acid Bacillus (*Lactobacillus sporogenes*)	Not specified				Strain not mentioned

Many products provide no strain-level details, undermining efficacy and safety assessments. Generic labels like “Lactic Acid Bacillus” cannot substitute for well-characterized strains such as *L. rhamnosus* ATCC 53103.^32^ Contaminants like *E. faecium*, a cause of nosocomial infections, pose further risks, especially to immunocompromised individuals.^33^

Probiotic strains may carry transferable antibiotic resistance genes,^22^^,^^33^ and underreporting of adverse effects in trials hinders risk evaluation.^34^ Collectively, these findings underscore the need for strict strain identification, robust quality control, and mandatory, genomics-informed premarket safety review to strengthen probiotic safety oversight while remaining operationally feasible.^30^^,^^44^

## Strain-specific efficacy and safety concerns in Indian probiotic products

Randomized controlled trials (RCTs) underline the therapeutic potential of select probiotic strains marketed in India. For instance, *S. boulardii* CNCM I-745 (Econorm) significantly enhanced Helicobacter pylori eradication rates (86.0% vs. 74.7%, *p* = 0.02) and reduced antibiotic-associated diarrhea (AAD) (2.0% vs. 46.4%, *p* = 0.02), while improving treatment adherence (95.0% vs. 91.2%, *p* < 0.001).^37^ Similarly, CNCM I-3799 (Ecoviva, Sporit GG) shortened pediatric acute diarrhea duration (65.8 vs. 95.3 hours, *p* = 0.0001) without adverse events, highlighting its resilience and safety.^45^

Other clinically supported strains include *B. clausii* UBBC-07 (Tufpro), which decreased diarrhea duration in Indian children (75.66 vs. 81.6 hours, *p* < 0.05),^46^ and *L. rhamnosus* ATCC 53103 (Sporit GG, Sporlac GG), which significantly reduced infantile colic (104 vs. 242 minutes, *p* < 0.001) and increased gut Lactobacillus levels (*p* = 0.048).^47^ *B. subtilis* HU58 in Darolac also aided AAD recovery.^48^ The three-strain blend in Bifilac (*S. faecalis* T-110, *C. butyricum* TO-A, and *B. mesentericus* TO-A) lowered serum phosphate in hemodialysis patients.^49^

However, concerns persist around generically labeled products such as Nutrolin B, Sporlac, and Gnorm, which cite outdated nomenclature (“Lactic Acid Bacillus”) without strain-level identity or clinical validation. These products often lack RCT backing and have shown contamination risks. For example, whole-genome sequencing has identified antimicrobial resistance determinants with predicted mobilization potential, including tet(L), catA10, and ant(4′)-Ib, in some *Lactobacillus sporogenes* (*Bacillus coagulans*) formulations, raising concerns regarding their quality and reliability.^48^^,^^49^

## Regulatory and scientific pathways forward

In this study, probiotic efficacy was defined operationally as the capacity of a product to deliver viable microorganisms at doses supported by clinical evidence, to survive gastrointestinal transit, and to retain functional viability during concurrent antibiotic exposure.^50^^,^^51^ Probiotic efficacy is well established to be strain-, dose-, and formulation-specific, with clinical benefit demonstrated only for selected organisms and indications rather than for probiotics as a class.^52-54^ Reported effective doses commonly range from 10⁹–10¹⁰ CFU per dose, although requirements vary by strain; for example, *S. boulardii* CNCM I-745 has demonstrated efficacy at approximately 5 × 10⁹ CFU (~250 mg) administered twice daily.^55^ Accordingly, the present work does not assess clinical efficacy per se, but evaluates microbiological and genomic attributes that constitute prerequisites for efficacy, as emphasized in international guidelines and recent comprehensive reviews.^11^^,^^55^

To enhance probiotic quality and clinical reliability, key strategies include enforcing strain-specific labeling using validated nomenclature and genomic confirmation to ensure accurate identification and efficacy. Premarket testing should be mandatory, including standardized *in vitro* assays and genomic screening for CFU counts, antimicrobial resistance genes, and virulence factors to improve quality control. Implementing strong postmarket surveillance can help track adverse events, monitor product stability, and assess real-world performance. Lastly, adopting internationally harmonized standards would ensure consistency across markets, boosting consumer confidence and the scientific credibility of the global probiotic industry.

## Conclusion

This study underscores the pressing need to address the pervasive issues of generic labeling, taxonomic inaccuracies, and inconsistent quality in commercial probiotics in India. While certain bacterial strains, such as *B. clausii,* offer clinical benefits, their safety and reliability are undermined by strain misidentification and antibiotic resistance concerns. In contrast, *S. boulardii* (Econorm) demonstrated consistent labeling, superior GI survivability, antibiotic resilience, and an absence of transferable resistance or virulence genes, establishing it as the most clinically sound option for acute diarrhea management.

To ensure patient safety and therapeutic efficacy, India may have to adopt more stringent regulatory practices, including mandatory strain verification and quality assurance protocols. Addressing these gaps is critical not only for improving health outcomes but also for fostering trust in probiotics as a scientifically validated therapeutic category.

## Limitations

This study assessed 13 widely used probiotic formulations in India, including multiple manufacturing batches, enabling evaluation of product- and batch-level variability. Probiotic viability was measured at a single time point per batch, without assessment of shelf-life stability or temporal changes during storage. Future studies incorporating a larger number of formulations, longitudinal stability analyses, and expanded market coverage will be important to strengthen population-level inference. The genomic context of detected antimicrobial resistance genes (e.g., adjacent insertion sequences or transposases) was not assessed and requires further investigation to determine their mobility potential. Also, the gastrointestinal simulation was comparative and excluded control strains due to the lack of established standards; inclusion of validated controls in future studies would strengthen model validation. Despite these limitations, the findings, particularly regarding labeling accuracy, genomic safety, and the consistent performance of *S. boulardii* highlight the need for broader surveillance and strengthened regulatory oversight.

## Supplementary Material

Supplementary Material Supplementary_Table_1.docx

## Data Availability

Genome sequencing data are available in NCBI under BioProject PRJNA1283376.

## Patient and public involvement

NA.
