# Supplementary material for: Safety and efficacy concerns in Indian probiotics: Insights from whole-genome sequencing and in vitro assessment
Source: Gut Microbes Rep. 2026 May 14;3(1):2668863. doi: 10.1080/29933935.2026.2668863 (PMC13178174; doi:10.1080/29933935.2026.2668863)
Supplement: Supplementary Material — Supplementary_Table_1.docx [file KGMR_A_2668863_SM1430.docx]

**Supplementary Table1: Predicted horizontal gene transfer potential of genes detected in probiotic isolates:** Mobility classification was based on published evidence of gene localization (plasmid vs chromosomal), association with mobile genetic elements, and the presence of insertion sequences detected in this study (Fig. 3). Genes reported on plasmids or transposons were classified as high mobility, those occasionally linked to mobile elements as moderate mobility, and chromosomally encoded intrinsic genes as low mobility.

| **Gene** | **Resistance mechanism** | **Literature evidence for mobility** | **Predicted mobilizability** |
| --- | --- | --- | --- |
| tet(L) | Tetracycline efflux transporter | Frequently plasmid- or transposon-associated in Gram-positive bacteria [1,2] | Detected in Bifilac (*B. mesentericus*, *B. coagulans*); IS elements present (IS1182, IS200/IS605, IS21, IS3, IS30). Co-occurrence suggests moderate–high mobilization potential. |
| erm(34) | 23S rRNA methyltransferase | Chromosomally encoded in *Bacillus clausii* [3] | Detected in Enterogermina and Tufpro (*B. clausii*); IS elements present, but literature suggests low mobilization potential. |
| blaBCL-1 | Class A β-lactamase | Intrinsic β-lactam resistance determinant in *B. clausii* [4] | Detected in *B. clausii* isolates; IS elements present, but predicted low mobility intrinsic determinant. |
| mphK | Macrolide phosphotransferase | Antibiotic-modifying enzymes sometimes associated with mobile elements [5] | Detected in Bifilac and Darolac Bacillus isolates; IS elements present. Predicted moderate mobilization potential. |
| aadK | Aminoglycoside adenylyltransferase | Aminoglycoside resistance enzymes frequently occur in transferable resistance gene pools [5] | Observed in Bacillus isolates; IS elements present. Predicted moderate mobilization potential. |
| ant(4′)-Ib | Aminoglycoside nucleotidyltransferase | Often plasmid-associated in Gram-positive bacteria [5] | Detected in *B. clausii* isolates; IS elements present. Predicted moderate–high mobilization potential. |
| satA_Bs | Streptothricin acetyltransferase | Often chromosomal in *Bacillus subtilis* [6] | Detected in Bifilac and Darolac isolates; IS elements present. Predicted low–moderate mobilization potential. |
| rphC | Rifamycin phosphotransferase | Rarely associated with mobile elements [7] | Observed in Bacillus isolates; IS elements present. Predicted low mobility. |
| vmlR | ABC-F ribosomal protection protein | Intrinsic ribosomal protection protein in Bacillus [8] | Detected in Bifilac and Darolac isolates; IS elements present, but predicted low mobility. |
| catA10 | Chloramphenicol acetyltransferase | Frequently plasmid- or transposon-associated [1] | Detected in *B. clausii* isolates; IS elements present. Predicted moderate–high mobilization potential. |
| clbB | rRNA modification enzyme | Typically chromosomal [9] | Detected in *B. clausii* isolates; IS elements present, but predicted low mobility. |
| hngB | Hemolysin-associated gene | Virulence genes generally chromosomal [10] | Detected in Bifilac (*B. mesentericus*); IS elements present, but predicted low mobility. |
| bslA / yugB | Biofilm matrix protein | Core chromosomal biofilm gene in *Bacillus subtilis* [11] | Detected in Darolac (*B. subtilis*); IS elements present, but predicted low mobility. |

**Table Reference**

1. Mathur S, Singh R. Antibiotic resistance in food lactic acid bacteria—a review. International journal of food microbiology. 2005 Dec 15;105(3):281-95.
2. Gueimonde M, Sánchez B, G. de los Reyes-Gavilán C, Margolles A. Antibiotic resistance in probiotic bacteria. Frontiers in microbiology. 2013 Jul 18;4:202.
3. Bozdogan B, Galopin S, Leclercq R. Characterization of a new erm-related macrolide resistance gene present in probiotic strains of Bacillus clausii. Applied and environmental microbiology. 2004 Jan;70(1):280-4.
4. Khatri I, Sharma G, Subramanian S. Composite genome sequence of Bacillus clausii, a probiotic commercially available as Enterogermina®, and insights into its probiotic properties. BMC microbiology. 2019 Dec 30;19(1):307.
5. Wright GD. Bacterial resistance to antibiotics: enzymatic degradation and modification. Advanced drug delivery reviews. 2005 Jul 29;57(10):1451-70.
6. Burckhardt RM, Escalante-Semerena JC. In Bacillus subtilis, the SatA (formerly YyaR) acetyltransferase detoxifies streptothricin via lysine acetylation. Applied and Environmental Microbiology. 2017 Nov 1;83(21):e01590-17.
7. Goldstein BP. Resistance to rifampicin: a review. The Journal of antibiotics. 2014 Sep;67(9):625-30.
8. Sharkey LK, O’Neill AJ. Antibiotic resistance ABC-F proteins: bringing target protection into the limelight. ACS Infectious Diseases. 2018 Jan 27;4(3):239-46.
9. Wilson DN. Ribosome-targeting antibiotics and mechanisms of bacterial resistance. Nature reviews microbiology. 2014 Jan;12(1):35-48.
10. Navarre WW, Schneewind O. Surface proteins of gram-positive bacteria and mechanisms of their targeting to the cell wall envelope. Microbiology and molecular biology reviews. 1999 Mar 1;63(1):174-229.
11. Hobley L, Ostrowski A, Rao FV, Bromley KM, Porter M, Prescott AR, MacPhee CE, Van Aalten DM, Stanley-Wall NR. BslA is a self-assembling bacterial hydrophobin that coats the Bacillus subtilis biofilm. Proceedings of the National Academy of Sciences. 2013 Aug 13;110(33):13600-5.
